# Work Ethics and Ethical Attitudes among Healthcare Professionals: The Role of Leadership Skills in Determining Ethics Construct and Professional Behaviors

**DOI:** 10.3390/healthcare10081399

**Published:** 2022-07-27

**Authors:** Fouad Sakr, Chadia Haddad, Rony M. Zeenny, Hala Sacre, Marwan Akel, Katia Iskandar, Aline Hajj, Pascale Salameh

**Affiliations:** 1 School of Pharmacy, Lebanese International University, Beirut 1105, Lebanon; marwan.akel@liu.edu.lb (M.A.); katia_iskandar@hotmail.com (K.I.); 2 Institut Mondor de Recherche Biomédicale, UMR U955 INSERM, Université Paris-Est Créteil, 94010 Créteil, France; 3 INSPECT-LB (Institut National de Santé Publique, d’Épidémiologie Clinique et de Toxicologie-Liban), Beirut 1103, Lebanon; chadia_9@hotmail.com (C.H.); rz37@aub.edu.lb (R.M.Z.); halasacre@hotmail.com (H.S.); pascalesalameh1@hotmail.com (P.S.); 4 Research Department, Psychiatric Hospital of the Cross, Jal El Dib 1201, Lebanon; 5 School of Health Sciences, Modern University of Business and Science, Beirut 1103, Lebanon; 6 Department of Pharmacy, American University of Beirut Medical Center, Beirut 1105, Lebanon; 7 Faculty of Pharmacy, Lebanese University, Hadath 1103, Lebanon; 8 Laboratoire de Pharmacologie, Pharmacie Clinique et Contrôle de Qualité des Médicament, Saint-Joseph University, Beirut 1103, Lebanon; aline.hajj@usj.edu.lb; 9 Faculty of Pharmacy, Saint-Joseph University, Beirut 1103, Lebanon; 10 School of Medicine, Lebanese American University, Byblos 1401, Lebanon; 11 Department of Primary Care and Population Health, University of Nicosia Medical School, Nicosia 2408, Cyprus

**Keywords:** healthcare, ethics, leadership, professional behavior, ethic construct, intrinsic work motivation

## Abstract

(1) Background: The provision of healthcare is transforming, necessitating changes in descriptions and frameworks for ethical leadership. This study aimed to assess the association of the different leadership skills with the work ethical constructs and attitudes among healthcare professionals. (2) Methods: A cross-sectional study design using a snowball sampling technique was used to enroll healthcare practitioners. The questionnaire used in this study included two parts: the first part gathered the sociodemographic characteristics of the participants, while the second part consisted of three validated assessment scales, i.e., work ethics, ethical attitudes questionnaire for public health professionals, and leadership skills questionnaire. (3) Results: Higher work ethics and a higher intrinsic work motivation subscale were significantly associated with high leadership administrative skills (Beta = 6.04, *p* = 0.019, and Beta = 2.55, *p* < 0.001, respectively). However, higher leadership conceptual skills (Beta = −1.07, *p* = 0.027) were associated with a lower intrinsic work motivation subscale. Higher leadership administrative skills (Beta = 28.39, *p* < 0.001) were significantly associated with higher ethical attitudes scores. (4) Conclusions: Higher administrative leadership has an imperative positive impact on work ethic in the provision of different public health services. Leadership skills are not limited to a specific profession, experience, or career stage of health care, and could significantly predict the individual ethical attitude and professional behavior.

## 1. Introduction

Ethical behaviors are a crucial element of health care and are fundamental to providing a high quality of patient services [1,2]. Standards of health care ethics aim to define how professionals should act when providing patient care [3,4]. In general, professional ethic is described as “a set of beliefs and attitudes reflecting the fundamental value of work” and is classified as an individual difference construct [5]. Work ethics reveal the values of an individual’s behavior, commitments, and performance when treating patients [1]. It is a collection of attitudes and behaviors at work rather than a single concept. Miller et al. characterize work ethic as (a) multi-dimensional; (b) pertains to work, and work-related activity in general, not specific to any particular job; (c) learned; (d) refers to attitude and beliefs; (e) a motivational construct reflected in behavior; (f) secular, not necessarily tied to any one set of religious beliefs [6]. In this study, all ethical aspects and dimensions are considered constructs.

The different dimensions of work ethics diverge through career stages [7]. Several studies differentiated work ethic based on professional behavior in the context of patient care, determining ethical issues relating to patient communication and societal disparities [8,9,10]. Additionally, healthcare ethics were reported to be marginally pertinent to professional behaviors that appear to be better controlled by personal and organizational morals [11,12]. In effect, an ethical climate can impact corporate ideals and hence encourage moral behavior among personnel. A literature review reveals that an ethical work environment is one of the most essential aspects that affects decision-making and behavior [13]. The provision of health care has been traditionally cherished in codes of ethics and governed by morals and professional behaviors. Several factors impact ethical behaviors in clinical practice and the value of codes linking to the healthcare setting, practice proficiency, moral education, institutional context, and accepted social norms [14]. Indeed, ethical standards pertain to nearly identical norms and patient care devotions, while each healthcare profession characterizes its principles [15]. Public health professionals are dedicated to holistic personal or community care rather than to the medical condition or classification [14,16], although the principles of care may vary from one discipline to another within the health care setting [17].

The provision of healthcare is transforming, necessitating changes in descriptions and frameworks for ethical leadership. Researchers and practitioners are increasingly providing much greater attention to leadership skills. According to Northouse, a core model of leadership embraces administrative, interpersonal, and conceptual leadership skills. Administrative leadership skills include showing technical competence and managing people and resources. Interpersonal leadership skills primarily involve handling interpersonal conflicts, demonstrating emotional intelligence, and being socially perceptive. Finally, conceptual leadership skills necessitate creating visions, strategic planning, and problem-solving [18]. Conventional ethical leadership has focused on professional procedures and attitudes, particularly at the level of the provider–patient interaction. Although healthcare is widely becoming team-based, and health systems are progressively comprising principles of public health, individual practitioner performance in specific clinical interactions continues to be ethically relevant. In the end, even secluded examples of misconduct or misbehavior can jeopardize trust in the public health system [19].

Various research papers have examined and practically evaluated the value of work ethic [20,21,22,23]. The association between work ethic, on the one hand, and attitudes and behaviors, on the other hand, emphasizes the importance of the work ethic construct. For instance, individuals with strong moral beliefs are more devoted, fulfilled, and engaged in their professions. While previous literature aimed to determine the differences in professional behavior between generations and career stages, it did not evaluate the determinants of the healthcare ethic construct and the predictors of professional healthcare behavior. Moreover, limited evidence is available around the role of leadership in shaping work ethic and professional behaviors in the practice of public health. This study aimed to determine the role of leadership in healthcare ethics and the association of the different leadership skills with the work ethic construct and ethical behavior among healthcare professionals.

## 2. Materials and Methods

### 2.1. Study Design and Sampling

A cross-sectional online survey among 245 healthcare professionals was conducted in Lebanon between July and December 2021. The questionnaire, created on Google forms, was distributed on social media to healthcare facilities to collect data (universities, hospitals, pharmaceutical industry, and others), using the snowball sampling technique. All healthcare practitioners who were over 18 and had access to the internet were eligible. The participants received no compensation in exchange for their participation, which was voluntary and anonymous.

### 2.2. Ethics Approval and Consent to Participate

The Research and Ethics Committee of the School of Pharmacy at the Lebanese International University approved the study protocol (2020RC-046-LIUSOP). The participants were informed of the study objectives on the landing page of the online questionnaire and gave their consent before enrolling.

### 2.3. Questionnaire

The questionnaire was in English and included two sections: the first section gathered the sociodemographic and work characteristics of the participants (such as age, gender, marital status, monthly income, the highest level of education completed, the country where the highest degree was earned, type of healthcare organization, profession, years of experience, and work position); the second section included the following assessment validated scales:

#### 2.3.1. Work Ethics

This 10-item self-reported scale was developed and validated to measure the attitude and belief of the respondents toward work ethics [24]. Three parameters (Work as Central Life Interest, Moral Approach to Work, and Intrinsic Work Motivation) are derived from the scale that covers most dimensions of the contemporary work ethic construct. All the items were phrased positively, and the responses were collected on a 4-point scale ranging from 0 (strongly disagree) to 3 (strongly agree) [24]. Permission to use the scale was obtained from the author of the article. The Cronbach’s alpha value for the total scale was 0.869.

#### 2.3.2. Ethical Attitudes Questionnaire for Public Health Professionals

This 33-item scale was initially developed to evaluate the general (24 items) and specific (9 items) ethical attitudes of public health professionals [14]. In this study, the 24-item subset that assessed the general ethical attitudes was used among all healthcare practitioners. All the items were rated from 0 (I almost never consider this attitude) to 5 (I always consider the attitude) [14]. Higher scores indicated a high ethical attitude. The Cronbach’s alpha value for the total scale was 0.983.

#### 2.3.3. Leadership Skills Questionnaire

The leadership skills questionnaire consists of 18 items, which are divided into three categories of leadership skills: administrative, interpersonal, and intellectual [18]. All the items were rated on a 5-point Likert scale ranging from 1 (not true) to 5 (very true). The three types of leadership were calculated independently, and each one was divided into two categories: high (scores ≥ 16) and low (scores < 16). In this study, the Cronbach’s alpha values for the three types of leadership were 0.882, 0.894, and 0.906 for leadership administrative, interpersonal, and conceptual skills, respectively. 

### 2.4. Statistical Analysis

The data were analyzed on SPSS software version 25. A descriptive analysis was carried out using counts and percentages for categorical variables and means and standard deviations for continuous measures. The sample was normally distributed, as verified by a visual inspection of the histogram, and the skewness and kurtosis were below |1.96| [25]. Moreover, the normality of the ethical attitudes total scale was verified by the normality line of the regression plot and the scatter plot of the residual. After checking for the normality, the independent-sample t-test was used to compare the mean of the work ethics scale, subscales, and the ethical attitudes total scale between two groups, while the ANOVA test was used to compare three or more means.

A multivariate analysis of covariance (MANCOVA) was carried out to compare the work ethics scale and subscales between the leadership groups (high vs. low leadership skills), taking into account potential confounding variables, i.e., age, gender, education level, and years of experience. A linear regression analysis was performed, taking the ethical attitudes total scale as the dependent variable. All the variables that showed a *p*-value < 0.2 in the bivariate analysis were included in the model to eliminate potential confounding factors. A *p*-value < 0.05 was considered significant.

## 3. Results

### 3.1. Sample Description

Table 1 shows the sociodemographic and other characteristics of the participants. The majority of the participants were female (62.0%); 52.2% were single; 58.8% had a high income; and 44.5% had a doctorate or a Master’s degree. Most of the participants earned their degree from Lebanon (87.8%); 80% were working in a healthcare organization; and 75.9% were in the private sector. More than half of the participants were pharmacists (59.2%); 69.0% worked full-time; and 24.9% had between 6 and 10 years of working experience. The mean age of the participants was 34.02 ± 9.20 years.

### 3.2. Description of the Ethics Scales Used

Table 2 describes the median, mean, SD, and range of the ethics scales that were used in this study. The mean work ethics scale was 23.34 ± 5.06, with a median of 24. The mean ethical attitudes scale was 82.15 ± 39.68, with a median of 98.

### 3.3. Bivariate Analysis

Table 3 shows the bivariate analysis, taking the work ethics total scale, subscales, and the ethical attitude scale as the dependent variables. Significantly higher means of the work ethics scale, subscales, and ethical attitude scale were found among those with high administrative, interpersonal, and conceptual leadership, compared to those with low leadership skills. Moreover, females had significantly higher means of the work ethics scale, work as central life interest subscale, and ethical attitude scale, than males. Compared to those with other degrees, participants with a doctorate had higher means of moral approach to work scores and intrinsic work motivation scores. Moreover, a higher mean of the moral approach to work subscale was found among those who had working experience of more than ten years, compared to the groups with less than 5 years and between 6 and 10 years of working experience.

### 3.4. Multivariable Analysis

The MANCOVA analysis was performed, taking the work ethics total scale and subscales as the dependent variables and the three leadership skills as the independent variable after adjusting for the covariates (age, gender, education level, and years of experience).

Higher administrative leadership skills were significantly associated with higher work ethics (Beta = 6.04) and a higher intrinsic work motivation subscale (Beta = 2.55). However, higher conceptual leadership skills (Beta = −1.07) were associated with a lower intrinsic work motivation score.

No significant association was found between leadership skills and work as a central life interest, and moral approach to work subscales (*p* > 0.05 for all). The multivariable analysis of covariance is shown in Table 4.

**Figure 1:**
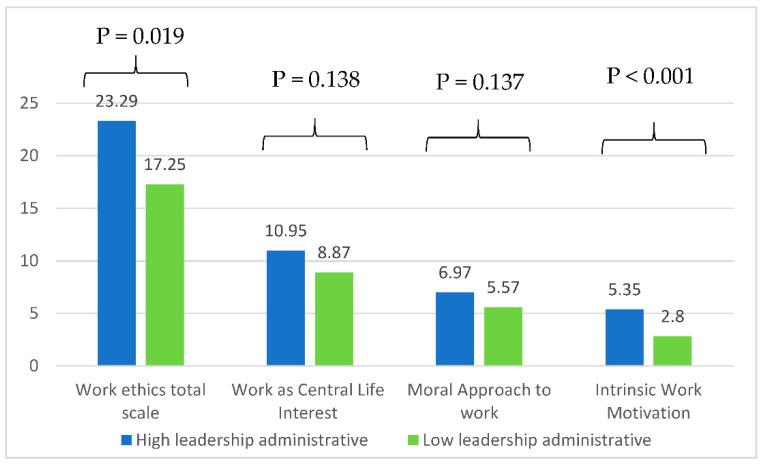
Mean values of the work ethics scale according to high/low administrative leadership skills adjusted for age, gender, education level, and years of experience.

**Figure 2:**
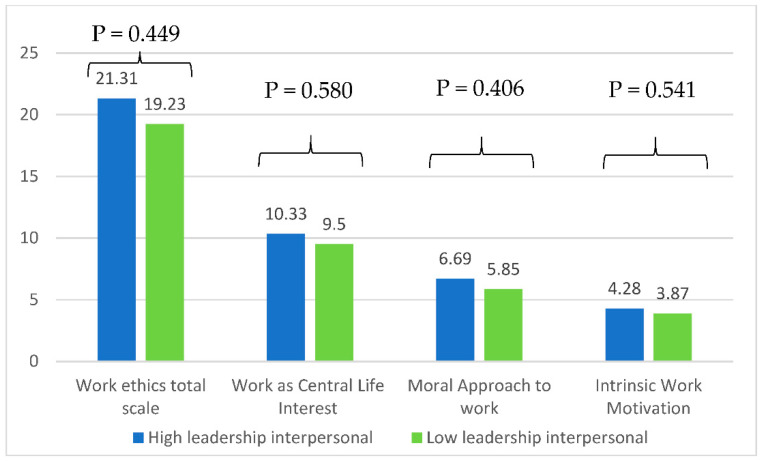
Mean values of the work ethics scale according to high/low interpersonal leadership skills adjusted for age, gender, education level, and years of experience.

**Figure 3:**
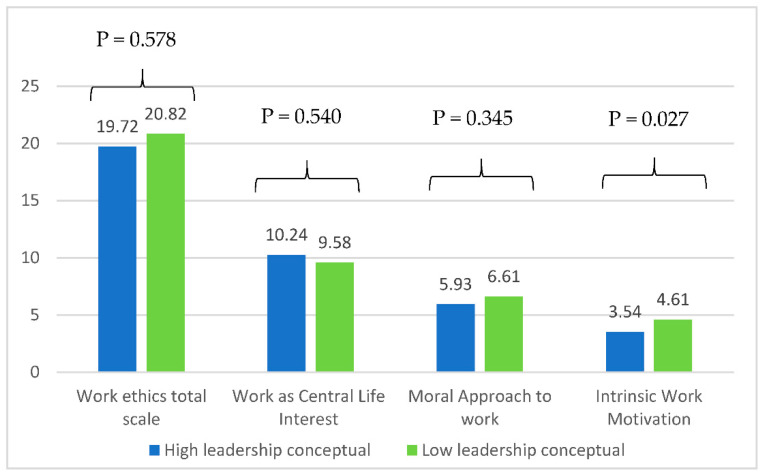
Mean values of the work ethics scale according to high/low conceptual leadership skills adjusted for age, gender, education level, and years of experience.

Figure 1, Figure 2 and Figure 3 show the means of the work ethics total scale and subscales between high and low leadership skills after adjustment over age, gender, education level, and years of experience. Significantly higher means of the work ethics total scale and intrinsic work motivation subscale were found among those with high administrative leadership skills as compared to those with low leadership (Figure 1).

No significant difference was found between the work ethics total scale and subscales and those with high and low interpersonal leadership (*p* > 0.05 for all) (Figure 2).

A significantly lower mean of the intrinsic work motivation subscale was found among those with high conceptual leadership skills as compared to those with lower leadership skills. No significant association was found between the other ethic scales and leadership skills (Figure 3).

A linear regression model was performed, taking the ethical attitudes total scale as the dependent variable. The results showed that higher administrative leadership skills (Beta = 28.39) were significantly associated with a higher ethical attitudes scale (Table 5).

## 4. Discussion

The current study evaluated the leadership skill determinants of ethics in providing health care with the overall work ethic construct and its components: central life interest, moral approach to work, and intrinsic work motivation. It also determined the leadership predicting skills of ethical attitudes. Higher administrative leadership skills were significantly associated with higher work ethics, higher intrinsic work motivation, and better ethical attitudes. However, a significant inverse association was found between intrinsic work motivation and conceptual leadership skills. To the best of our knowledge, there are no established principles on how leadership skills can impact the different dimensions of ethics in the provision of healthcare and how it can influence the professional attitudes of health professionals. 

Our results showed higher means of overall work ethics among health professionals with high administrative leadership skills. Previous research determined that work ethic positively influences the fit of an individual in an institute [26]. Nevertheless, no studies have previously examined the determinants of the work ethic construct among healthcare practitioners, while it is assumed that every provider should have a work ethic to practice. The principal dimensions of work ethic in other disciplines other than health are reportedly influenced by individual moral reasoning and how the workplace operates ethically based on real-life experiences [27]. In any particular work, administrative skills involve planning competencies, work organization, activity coordination, and assigning tasks to people adequately [28]. In the current study, the positive association between administrative skills and exemplary values in providing healthcare could map leadership with the vast construct of work ethics on the promotion of health and prevention of sickness, reduction in health risks, research of epidemiology, public health features, and socioeconomic inequities in health status [29].

High leadership administrative skills were significantly associated with higher intrinsic work motivation. Intrinsic motivation is defined as the desire to engage in conduct that is innately appealing or gratifying [30,31]. Janssen and colleagues determined that specific intrinsic work motivation among nurses is primarily driven by challenging and desirably diverse job skills, autonomy, social interactions, and learning opportunities [32]. Our findings refine insight into work ethic relationships, suggesting that administrative leadership skills could enhance the job content quality and thus contribute to the ethical construct through the intrinsic work motivation module. Moreover, if stakeholders aim to improve intrinsic work motivation, they should focus on building leadership capacities among health care professionals. 

Conceptual leadership skills in this study were inversely associated with intrinsic work motivation. While administrative skills are concerned with managing work, conceptual skills are concerned with cognitive features of leadership and are decisive for organizational vision and strategic planning [18]. Previous literature indicated that both healthcare staff and managers experience ethics-related distress, and healthcare leaders are more concerned with norms upkeep and job performance rather than demonstrating leadership when challenged with ethical issues [33,34,35]. In this study, healthcare providers with high leadership conceptual skills had significantly lower means of intrinsic work motivation. Work exhaustion and burnout, resulting from workload, limited resources and support, and lack of time, could explain this finding [36,37]. However, evidence to support this relationship within the work ethic construct remains insufficient. Further research is suggested in this context to determine the mediating effect of work exhaustion and burnout on conceptual leadership and intrinsic work motivation.

Our findings showed that high administrative leadership skills were significantly associated with higher professional ethical attitudes. Hariharan and colleagues suggested that healthcare practitioners who are more frequently in contact with patients experience more ethical challenges that could influence their attitudes. They also determined that experience at work is more important than ethics knowledge for professional behavior [38]. Those findings are not supported by our results, where no significant associations were found between professional behaviors and the years of working experience, nor the level of education. Previous literature conceived that ethical behavior is paramount in healthcare, as it is the core value of professionalism among practitioners. Healthcare providers must be ready for public exposure in terms of professionalism concerning ethical challenges [39,40]. The present study advocates that administrative leadership empowers health care professionals to manage at all levels within a team. It also proposes that higher leadership capacities positively associate with professional maturity, and thus with moral reasoning and behavior in professional practices. 

### 4.1. Implications for Practice

Research on health ethics converges on identifying models and tenets that have been established and advanced by professionals and lead practitioners over an extended period [29]. Leadership could prominently shape these values to construct a work ethic in health care. The authors view leadership as a broad definition of competencies that apply to every healthcare professional and are not restricted to managerial positions. The present study reveals that higher leadership skills have an imperative positive impact on work ethics and professional behaviors in the provision of different healthcare services. Leadership skills are not limited to a specific profession, experience, or career stage of healthcare and could significantly predict individual professional attitude and behavior. The complexity of the healthcare system requires every practitioner to be a leader in providing health services and prioritizing work ethic as a core value. Healthcare stakeholders should support establishing and progressing leadership capacities at an individual professional level, as they determine the overall breadth of work ethic construct and the intrinsic work motivation.

### 4.2. Limitations

This study has a few limitations. The cross-sectional design does not establish temporality; therefore, no causality can be determined. In addition, the measurement of the work ethic construct was unidimensional; however, the consequences of this limitation are believed to be reduced as the utilized assessment scale is a valid and reliable tool that covers all aspects of the work ethic construct. Moreover, this scale is a generic tool that has never been validated among healthcare professionals. Other research studies are suggested in this scope to tailor and validate a work ethic scale for practice and research in health care. As with all online surveys, there is an overrepresentation of more educated participants and a gender bias (higher women/men ratio); however, given the nature of the analytical study, we have no reason to think that the results could have differed if the selection methods were different. This study involved health professionals from one country and included a relatively small sample size. Sample size and cultural variations may have a positive or negative impact on the results. Moreover, an information bias is suspected given the delicateness of the topic, and residual confounding cannot be ruled out. Further studies are suggested to minimize the current biases.

## 5. Conclusions

Administrative rather than conceptual leadership skills shape the work ethics of healthcare professionals and could contribute to the ethical construct. Better administrative leadership fosters professional attitudes and intrinsic work motivation. Higher leadership capacities positively associate with professional maturity and thus with moral reasoning and behavior in professional practices. They can also empower health care professionals to manage at all levels within a team. Consequently, the work ethics construct in healthcare must be redefined to cope with the dynamic transformational change of the health system based on continuous soft skills education and training.

## Figures and Tables

**Table 1:** Sociodemographic and work characteristics of the participants (N = 245).

Variable	N (%)
**Gender**	
Male	93 (38.0%)
Female	152 (62.0%)
**Marital status**	
Single/widowed/divorced	128 (52.2%)
Married	117 (47.8%)
**Monthly income**	
No income	22 (9.0%)
Low (<1,500,000 LL)	7 (2.9%)
Intermediate (1,500,000–3,000,000 LL)	72 (29.4%)
High (>3,000,000 LL)	144 (58.8%)
**Highest education level**	
Doctorate (PhD, DBA, DPT, etc.)	49 (20.0%)
Master’s degree (MBA, MPH, etc.)	60 (24.5%)
PharmD	55 (22.4%)
Bachelor’s degree	74 (30.2%)
High school	7 (2.9%)
**Highest diploma earned from**	
Lebanon	215 (87.8%)
Abroad	30 (12.2%)
**Currently working in a healthcare organization**	
Yes	196 (80.0%)
No	49 (20.0%)
Type of healthcare organization *	
Private sector	186 (75.9%)
Public sector	55 (22.4%)
Type of profession *	
Healthcare professionals with administrative position	5 (2.0%)
Nurse	13 (5.3%)
Pharmacist	145 (59.2%)
Physician	10 (4.1%)
Dentist	20 (8.2%)
Researcher	15 (6.1%)
Dietitian	2 (0.8%)
Physical therapist	15 (6.1%)
Healthcare professionals with social role	2 (0.8%)
Healthcare professionals with other specialties	18 (7.3%)
**Working experience**	
Less than 5 years	94 (38.4%)
6–10 years	61 (24.9%)
More than 10 years	85 (34.7%)
Does not apply	5 (2.0%)
**Type of work**	
Full time	169 (69.0%)
Part-time	56 (22.9%)
Does not apply	20 (8.2%)
	**Mean ± SD**
**Age in years**	34.02 ± 9.20

* The same person may have multiple responses in case of multiple professional roles.

**Table 2:** Description of the work ethics scale and professional ethical scale in the study.

	Median	Mean	SD	Minimum	Maximum
**Work ethics total scale**	24.00	23.34	5.06	5.00	30.00
Work as central life interest	12.00	11.49	2.70	2.00	15.00
Moral approach to work	7.00	6.94	1.79	1.00	9.00
Intrinsic work motivation	5.00	4.89	1.29	0	6.00
**Professional ethical attitudes total scale**	98.00	82.15	39.68	0	120.00

**Table 3:** Bivariate analysis taking the ethical scales and professional ethical attitude as the dependent variables.

	Work Ethics Total Scale	Work as Central Life Interest	Moral Approach to Work	Intrinsic Work Motivation	Professional Ethical Attitude
	Mean ± SD	Mean ± SD	Mean ± SD	Mean ± SD	Mean ± SD
**Gender**					
Male	22.27 ± 5.33	10.84 ± 2.78	6.67 ± 1.95	4.75 ± 1.29	92.77 ± 22.87
Female	23.99 ± 4.79	11.89 ± 2.58	7.11 ± 1.68	4.98 ± 1.29	99.45 ± 22.43
*p*-value	**0.010**	**0.003**	0.066	0.170	**0.040**
**Marital status**					
Single/Widowed/Divorced	23.18 ± 5.18	11.52 ± 2.73	6.86 ± 1.78	4.79 ± 1.35	96.90 ± 22.29
Married	23.51 ± 4.94	11.47 ± 2.68	7.03 ± 1.81	5.00 ± 1.21	96.71 ± 23.46
*p*-value	0.617	0.878	0.469	0.201	0.954
**Monthly income**					
No income	22.95 ± 4.68	11.54 ± 2.66	6.68 ± 1.70	4.72 ± 1.45	100.90 ± 18.76
Low (<1,500,000 LL)	20.85 ± 9.82	10.57 ± 4.92	6.28 ± 3.45	4.00 ± 2.00	93.66 ± 32.86
Intermediate (1,500,000–3,000,000 LL)	23.11 ± 4.95	11.36 ± 2.70	6.93 ± 1.73	4.81 ± 1.23	98.30 ± 23.08
High (>3,000,000 LL)	23.63 ± 4.88	11.60 ± 2.59	7.02 ± 1.74	5.00 ± 1.24	95.41 ± 22.84
*p*-value	0.486	0.747	0.634	0.169	0.693
**Highest Education level**					
Doctorate	24.24 ± 5.23	11.85 ± 2.73	7.38 ± 1.77	5.00 ± 1.41	96.95 ± 21.26
Master’s degree	23.30 ± 4.88	11.46 ± 2.49	6.85 ± 1.99	4.98 ± 1.20	97.18 ± 23.31
PharmD	23.90 ± 4.69	11.72 ± 2.66	7.12 ± 1.57	5.05 ± 1.07	103.34 ± 15.22
Bachelor’s degree	22.79 ± 5.20	11.24 ± 2.91	6.75 ± 1.69	4.79 ± 1.31	92.07 ± 26.25
High school	18.71 ± 4.82	10.14 ± 2.19	5.28 ± 1.97	3.28 ± 1.70	99.00 ± 27.15
*p*-value	0.061	0.454	**0.032**	**0.012**	0.164
**Highest diploma earned from**					
Lebanon	23.27 ± 5.14	11.47 ± 2.72	6.93 ± 1.78	4.86 ± 1.32	97.34 ± 22.48
Abroad	23.83 ± 4.48	11.63 ± 2.38	7.06 ± 1.92	5.13 ± 1.07	92.60 ± 25.18
*p*-value	0.572	0.771	0.698	0.288	0.349
**Working experience**					
Less than 5 years	19.40 ± 6.02	10.80 ± 2.58	4.80 ± 1.48	3.80 ± 2.48	70.66 ± 39.25
6–10 years	22.91 ± 5.08	11.38 ± 2.82	6.71 ± 1.73	4.81 ± 1.24	97.20 ± 20.66
More than 10 years	23.67 ± 5.35	11.81 ± 2.80	7.01 ± 1.81	4.83 ± 1.34	98.96 ± 22.59
Does not apply	23.81 ± 4.74	11.43 ± 2.51	7.28 ± 1.77	5.09 ± 1.20	95.82 ± 24.36
*p*-value	0.197	0.706	**0.008**	0.109	0.206
**Type of profession**					
Administrative	25.60 ± 3.91	12.60 ± 2.40	7.80 ± 0.83	5.20 ± 1.09	97.00 ± 10.51
Nurse	21.30 ± 6.06	10.61 ± 3.09	6.23 ± 2.16	4.46 ± 1.33	79.92 ± 32.75
Pharmacist	23.69 ± 4.94	11.69 ± 2.73	7.00 ± 1.73	5.00 ± 1.20	98.79 ± 21.72
Physician	22.90 ± 5.74	11.40 ± 2.98	7.00 ± 1.82	4.50 ± 1.64	94.22 ± 21.47
Dentist	24.85 ± 4.18	12.00 ± 2.24	7.70 ± 1.62	5.15 ± 0.98	100.20 ± 16.49
Researcher	22.40 ± 6.00	11.00 ± 2.59	6.93 ± 2.21	4.46 ± 2.06	81.60 ± 27.62
Dietitian	26.50 ± 3.53	14.00 ± 1.41	7.50 ± 2.12	5.00 ± 0.01	118.00 ± 2.82
Physical therapist	22.00 ± 5.59	10.46 ± 2.82	6.73 ± 2.05	4.80 ± 1.37	95.85 ± 24.77
Social worker	17.00 ± 11.31	9.00 ± 7.07	5.00 ± 1.41	3.00 ± 2.82	82.00 ± 49.49
Other specialty	22.16 ± 3.61	11.00 ± 1.90	6.27 ± 1.60	4.88 ± 1.07	104.00 ± 14.63
*p*-value	0.206	0.320	0.207	0.318	**0.046**
**Type of work**					
Full time	23.58 ± 4.82	11.56 ± 2.60	7.04 ± 1.70	4.97 ± 1.22	97.81 ± 22.41
Part time	22.39 ± 5.90	10.98 ± 3.12	6.73 ± 2.07	4.67 ± 1.40	92.72 ± 23.98
Does not apply	23.95 ± 4.33	12.35 ± 2.00	6.70 ± 1.75	4.90 ± 1.51	100.00 ± 22.29
*p*-value	0.267	0.127	0.428	0.344	0.341
**Leadership administrative skills**					
High	23.84 ± 4.62	11.72 ± 2.53	7.06 ± 1.72	5.05 ± 1.11	98.75 ± 21.46
Low	16.18 ± 5.79	8.25 ± 3.08	5.25 ± 1.98	2.68 ± 1.66	70.35 ± 24.60
*p*-value	**<0.001**	**<0.001**	**<0.001**	**<0.001**	**<0.001**
**Leadership interpersonal skills**					
High	23.74 ± 4.65	11.67 ± 2.53	7.04 ± 1.72	5.01 ± 1.15	98.20 ± 21.70
Low	15.58 ± 6.61	8.00 ± 3.54	5.00 ± 2.17	2.58 ± 1.72	69.70 ± 27.57
*p*-value	**<0.001**	**<0.001**	**<0.001**	**<0.001**	**<0.001**
**Leadership conceptual skills**					
High	23.63 ± 4.78	11.66 ± 2.56	7.00 ± 1.74	4.97 ± 1.22	98.07 ± 22.03
Low	17.58 ± 6.94	8.33 ± 3.44	5.75 ± 2.34	3.50 ± 1.78	74.63 ± 25.44
*p*-value	**<0.001**	**<0.001**	**0.018**	**0.016**	**0.001**

Values marked in bold are significant.

**Table 4:** Multivariable analysis of covariance (MANCOVA).

	Beta	*p*-Value	Confidence Interval	
			Lower	Upper
**Work ethics total scale**				
Leadership administrative skills (high vs low *)	6.042	0.019	0.997	11.087
Leadership interpersonal skills (high vs low *)	2.082	0.449	−3.324	7.489
Leadership conceptual skills (high vs low *)	−1.101	0.578	−4.991	2.788
**Work as central life interest**				
Leadership administrative skills (high vs low *)	2.080	0.138	−0.671	4.831
Leadership interpersonal skills (high vs low *)	0.829	0.580	−2.120	3.777
Leadership conceptual skills (high vs low *)	0.660	0.540	−1.461	2.781
**Moral approach to work**				
Leadership administrative skills (high vs low *)	1.40	0.137	−0.452	3.267
Leadership interpersonal skills (high vs low *)	0.842	0.406	−1.151	2.835
Leadership conceptual skills (high vs low *)	−0.688	0.345	−2.122	0.745
**Intrinsic work motivation**				
Leadership administrative skills (high vs low *)	2.554	<0.001	1.320	3.788
Leadership interpersonal skills (high vs low *)	0.411	0.541	−0.911	1.734
Leadership conceptual skills (high vs low *)	−1.073	0.027	−2.025	−0.122

Note: In the global model, the independent variable is leadership skills. Covariates are age, gender, education level, and years of experience.

**Table 5:** Multivariable analysis.

Model 1: Linear Regression Taking the Professional Ethical Attitudes Total Scale as the Dependent Variable					
	Unstandardized Beta	Standardized Beta	*p*-Value	Confidence Interval	
				Lower Bound	Upper Bound
Leadership administrative skills (high vs low *)	28.39	0.315	<0.001	16.56	40.23

Variables entered: Sex, type of work (pharmacies vs other), administrative, leadership interpersonal, and leadership conceptual. * Reference group.

## Data Availability

The data presented in this study are available on request from the corresponding author on reasonable request.
